# Towards clinically interpretable machine learning in emergency surgery: feature importance and insights across clinical time points in abdominal pain cases

**DOI:** 10.1007/s00423-026-04098-z

**Published:** 2026-06-16

**Authors:** Jonas Henn, Simon Hatterscheidt, Svetozar Nesic, Sebastian Nowak, Wolfgang Block, Johannes Röttgen, Ingo Gräff, Jörg C. Kalff, Alois M. Sprinkart, Andreas Buness, Hanno Matthaei

**Affiliations:** 1 Department of General, Visceral, Vascular and Transplant Surgery, University Hospital Bonn, Bonn, Germany; 2 Bonn Surgical Technology Center (BOSTER), University Hospital Bonn, Bonn, Germany; 3 Core Unit for Bioinformatics Data Analysis, University Hospital Bonn, Bonn, Germany; 4 Department of Diagnostic and Interventional Radiology, University Hospital Bonn, Bonn, Germany; 5 Department of Radiotherapy and Radiation Oncology, University Hospital Bonn, Bonn, Germany; 6 Department of Neuroradiology, University Hospital Bonn, Bonn, Germany; 7 Department of Clinical Acute and Emergency Medicine, University Hospital Bonn, Bonn, Germany

**Keywords:** Acute abdominal pain, Emergency surgery, Decision support, Clinical decision-making, Machine learning, Explainable artificial intelligence, Artificial intelligence

## Abstract

**Purpose:**

Machine learning (ML) may support decision-making for acute abdominal pain (AAP), but limited interpretability hinders adoption. We evaluated a random-forest classifier for predicting urgent abdominal surgery and examined how predictive value and feature importance evolve across clinical timepoints.

**Methods:**

In this retrospective single-center study, we included adults presenting with AAP to the emergency department. The outcome was urgent abdominal surgery within 24 h. Features were grouped into stepwise sets. For each feature set, models were trained in 20 random 80/20 train–test splits with randomized hyperparameter search. Performance was summarized by AUC ROC and AUC PR, and interpretability by permutation importance and SHapley Additive exPlanations (SHAP).

**Results:**

Among 1,350 patients (median age 43 years, 682 (50.5%) females), 276 (20.4%) underwent urgent surgery. The final model achieved a median AUC ROC of 0.83. Discrimination increased stepwise from basic data (0.53), symptoms (0.61), pain history (0.66), vital signs (0.68), laboratory values (0.76), and physical examination (0.83). Computed tomography added only marginal improvement (0.83). Feature importance shifted from symptoms to vital signs and subsequently laboratory markers, particularly c-reactive Protein, white blood cell count, and prothrombin time, complemented by guarding. SHAP analyses confirmed these trends by consistently linking abnormal laboratory or clinical values to operative outcomes.

**Conclusion:**

Interpretable ML enables pre-imaging risk stratification for urgent surgery in AAP. Near-final discrimination is achieved after laboratory testing and physical examination, while computed tomography adds limited value at population level. Timepoint-specific feature contribution may facilitate integration of ML into surgical decision-making.

## Introduction

Artificial intelligence (AI) and machine learning (ML) are increasingly explored for clinical decision-making (CDM) in surgery and emergency medicine [1]. These methods promise improved diagnostic accuracy [2] and outcome prediction [3] by leveraging heterogeneous clinical data. Despite encouraging results, limited interpretability and transparency of predictions and incomplete consideration of temporal data remain major barriers to clinical adoption. Clinicians must be able to understand the rationale behind model predictions; otherwise, trust, reliance and integration into routine workflows are unlikely [4]. This is particularly critical in emergency settings, where rapid and transparent CDM is essential.

Acute abdominal pain (AAP) illustrates this challenge. As one of the most frequent and potentially life-threatening presentations in emergency departments, AAP requires timely differentiation between conditions manageable conservatively and those necessitating urgent surgery [5]. CDM depends on the integration of sequentially available information, including history, vital signs, laboratory values, physical examination, and imaging [5]. Previous studies have shown that ML models can predict urgent surgery or specific surgical conditions in AAP with high discriminatory performance [6]. Most approaches, however, still treat all predictors as if simultaneously available, neglecting the stepwise acquisition of information in emergency care. An exception is the study by Schipper et al. who demonstrated that model accuracy improved when information was added at different decision points in suspected appendicitis [7]. However, their approach lacked explainable feature attribution. In contrast, *Ma et al.* applied explainability to gangrenous cholecystitis, yet their analysis was restricted to a single timepoint [8].

To our knowledge, no study has yet addressed AAP in general while simultaneously considering sequential clinical timepoints and explainable predictions. This gap reflects the broader challenge of interpretability, a key barrier to clinical adoption. Both surgeons and patients emphasize transparency, safety, and explainability as prerequisites for AI-supported CDM [9, 10]. Without insight into which features drive predictions at specific time points, clinicians may remain hesitant to rely on algorithmic support. Bridging this gap requires models that not only predict accurately, but also provide clinically meaningful, timepoint-specific explanations. This study aimed to evaluate the performance of a random-forest classifier for urgent abdominal surgery in AAP while quantifying the incremental value and attribution of sequentially available feature sets. By linking predictive accuracy to clinically interpretable feature importance across diagnostic time points, we sought to enable workflow-aligned and trustworthy integration of ML into emergency surgical CDM.

## Materials and methods

### Study cohort

We conducted a retrospective single-center study of adult patients presenting with AAP to the emergency department of a tertiary care hospital between January 2020 and December 2021. All adult patients with the chief complaint “abdominal pain in adults” were eligible for inclusion. The primary outcome was whether an urgent abdominal operation was performed within 24 h of admission. This outcome was defined by the realized surgical decision and therefore reflects real-world CDM. We deliberately chose this endpoint to model clinical decision pathways and to support early triage as a diagnostic support approach, rather than to replace physician decision-making or to retrospectively evaluate the correctness of surgical indications. The determination of whether surgery was truly indicated (e.g., avoidable surgery or missed surgical disease) would require a reliable ground truth, which is difficult to establish retrospectively and often subject to interpretation and hindsight bias. Consequently, we did not assess whether surgery was retrospectively deemed avoidable or could have been delayed, as such judgments were not consistently available in retrospective data. Our outcome therefore represents clinical practice rather than an absolute gold standard of surgical necessity. Final diagnoses were extracted from discharge letters for hospitalized patients and from emergency department records for ambulatory cases. Diagnoses were categorized into clinically relevant groups adapted from the framework proposed by Lameris et al. [11].

### Feature extraction and preprocessing

Clinical features were manually extracted from emergency department reports. Metric variables included age, vital signs, and laboratory results; categorical features such as sex, symptoms, physical examination, and radiological findings were encoded as binary variables. To reflect the diagnostic workflow, variables were grouped into seven feature sets: 1_Basic, 2_Symptoms, 3_Pain History, 4_Vital Signs, 5_Laboratory Values, 6_Physical Examination, and 7_computed tomography (CT) (see Table 1). To explore the predictive potential of routinely available information before surgical involvement, we deliberately ordered laboratory results before physical examination in the stepwise feature sets. This setup did not reflect the exact clinical workflow but was chosen to evaluate how far prediction can proceed without qualified surgical examination. The CT feature set comprised typical surgical pathologies, coded as binary variables: free intra-abdominal air, free fluid, fat stranding, and bowel obstruction. Ultrasound findings were not included, as they were not consistently available in a standardized format and are highly operator dependent. Missing values were not imputed, as imputation might have introduced data leakage [12]. Instead, missingness was directly handled by the H2O random-forest implementation, which accommodated missing values within decision trees [13].

**Table 1:** Predictor features by sets

Feature set	Feature	Missing ratio (%)	Median or absolute frequency (*N*)	IQR or relative frequency (%)
1. Basic	Age (a)	0.0	44.0	29.0–62.0
	Female Sex	0.0	682	50.5
2. Symptoms	Fever	26.2	102	7.6
	Nausea	15.0	382	28.3
	Vomiting	12.4	288	21.3
	Diarrhea	12.5	164	12.1
	Dysuria	13.6	49	3.6
	Previous Surgery	1.2	609	45.1
3. Pain History	Days of Pain (d)	14.1	1.0	0.0–2.0
	RHR	15.3	308	22.8
	Epigastric	15.4	259	19.2
	LHR	15.3	220	16.3
	RLR	15.3	129	9.6
	Periumbilical	15.3	140	10.4
	LLR	15.3	108	8.0
	RIR	15.3	562	41.6
	Hypogastric	15.3	244	18.1
	LIR	15.3	396	29.3
4. Vital Signs	Temperature (°C)	5.9	36.4	36.0–36.8
	RRsys (mmHg)	14.3	136.0	124.0–152.0
	RRdia (mmHg)	14.4	82.0	72.5–91.0
	HR (/min)	12.4	84.0	74.0–96.0
	SpO_2_ (%)	12.6	98.0	97.0–99.0
5. Laboratory	Creatinine (mg/dL)	2.4	0.8	0.7–1.0
	Bilirubin (mg/dL)	16.6	0.5	0.3–0.7
	GGT (U/L)	15.8	24.0	14.0–48.0
	ALT (U/L)	5.4	22.0	15.0–33.0
	AST (U/L)	13.2	24.0	20.0–33.0
	ALP (U/L)	11.9	74.0	59.0–96.0
	Lipase (U/L)	14.4	29.0	21.8–40.0
	Amylase (U/L)	23.0	57.0	41.0–78.0
	CRP (mg/L)	1.6	5.1	1.3–27.4
	Quick (%)	5.0	95.0	83.0–105.0
	PTT (s)	6.8	24.0	22.0–26.0
	WBC (10^3^/µL)	1.1	9.4	7.2–12.5
	Hb (g/dL)	1.1	13.6	12.3–14.8
	Lactate (mmol/L)	36.4	1.4	1.1–1.9
6. Physical Examination	RHR	3.1	329	24.4
	Epigastric	3.3	283	20.1
	LHR	3.2	238	17.6
	RLR	3.2	239	17.7
	Periumbilical	3.2	219	16.2
	LLR	3.2	220	16.3
	RIR	3.2	566	41.9
	Hypogastric	3.3	303	22.4
	LIR	3.2	426	31.6
	Guarding	2.7	200	14.8
7. CT	Free Air	73.0	28	2.1
	Free Fluid	80.0	103	7.6
	Fat Stranding	88.4	144	10.7
	Obstruction	87.8	27	2.0
8. Outcome	Urgent Surgery	0.0	276	20.4

Binary features are displayed with absolute (n) and relative frequency (%), metric features are displayed with median and interquartile range. RHR: right hypochondriac region, LHR: left hypochondriac region, RLR: right lumbar region, LLR: left lumbar region, RIR: right iliac region, LIR: left iliac region, RRsys: systolic blood pressure, RRdia: diastolic blood pressure, SpO2: peripheral oxygen saturation, GGT: gamma-glutamyl transferase, ALT: alanine transaminase, AST: aspartate transaminase, ALP: alkaline phosphatase, CRP: c-reactive protein, Quick: prothrombin time, PTT: partial thromboplastin time, WBC: white blood cell count, Hb: Hemoglobin, CT: computed tomography

### Machine learning

All analyses were performed using Excel (v16.100.4, Microsoft, Redmond, USA), R (v4.5.0, R Foundation for Statistical Computing, Vienna, Austria), and RStudio (v2025.5.0.496, Posit Software, Boston, USA). Computations were executed on a server with 24 CPU cores and 256 GB memory. A custom R script (https://github.com/boster-hub/pain_model_rf) orchestrated preprocessing, feature set partitioning, data splitting, model training, and computation of relative feature importance and SHAP values. Random-forest classifiers were trained using the H2O framework [13], separately for each of the seven incremental feature sets, thereby reflecting the stepwise acquisition of clinical information in practice. Model training was repeated 20 times to minimize sampling bias and for each iteration, the dataset was randomly split into training (80%) and test (20%) sets. Within each training set, hyperparameters were optimized by 10-fold cross-validation with random grid search (ntrees, maximum depth, sample rate, and mtries). Among all candidate models, the best-performing configuration was selected based on area under the precision–recall curve (AUC PR) in the training folds and then evaluated on the independent test set.

### Model evaluation and interpretability

Model performance was assessed on the held-out test sets using the area under the receiver operating characteristic curve (AUC ROC) and AUC PR. Median values with interquartile ranges (IQR) were reported across all iterations. Relative feature importance was derived from H2O’s permutation-based metric. In addition, SHapley Additive exPlanations (SHAP) values were computed using H2O’s implementation of feature contribution analysis for tree-based models, providing interpretable, patient-level insights into model predictions.

## Results

### Study cohort

A total of 1,350 patients presenting with AAP were included. Median age was 43 years (IQR 29–64), and 682 (51%) were female. Overall, 276 patients (20.4%) underwent urgent abdominal surgery within 24 h. Baseline characteristics are summarized in Table 1. The cohort comprised a broad spectrum of diagnoses, including both surgical and non-surgical conditions, as shown in Table 2.

**Table 2:** Final diagnoses of the study cohort

Diagnosis	Absolute frequency (*N*)	Relative frequency (%)
Non-specific abdominal pain	402	29.8
Appendicitis	127	9.4
Obstipation	101	7.5
Hernia	94	7.0
Renal and urinary tract disorders	76	5.6
Diverticulitis	69	5.1
Gastrointestinal disorders	57	4.2
Cholecystitis	55	4.1
Other	54	4.0
Bowel obstruction / Ileus	49	3.6
Vascular / Bleeding	42	3.1
Cholecystolithiasis	41	3.0
Abscess	39	2.9
Gynecological disorders	37	2.7
Perforated viscus	25	1.9
Malignancy	25	1.9
Extra-abdominal disorders	17	1.3
Pancreatitis	15	1.1
Bowel ischemia	14	1.0
Inflammatory bowel disease	6	0.4
Cholangitis	5	0.4

Diagnoses were categorized into clinically relevant groups adapted from the framework proposed by Laméris et al. [11]

### Model performance

**Fig. 1:**
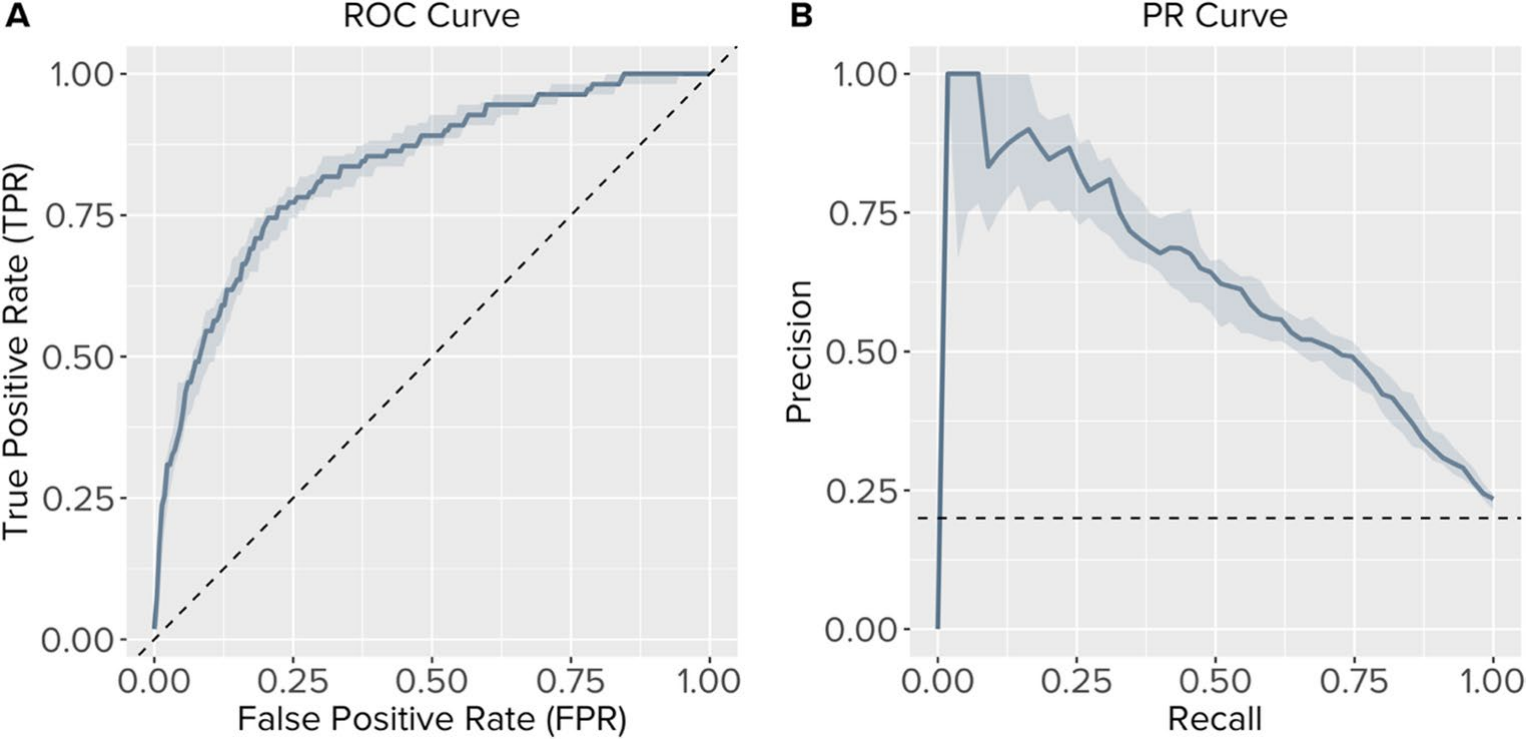
Performance of the final model (all feature sets). **A** Receiver operating characteristic (ROC) curve: Median and interquartile range (IQR) of the performance (blue) and chance level (no predictive value) as a reference (dashed line). **B** Same representation for the precision recall (PR) curve

**Fig. 2:**
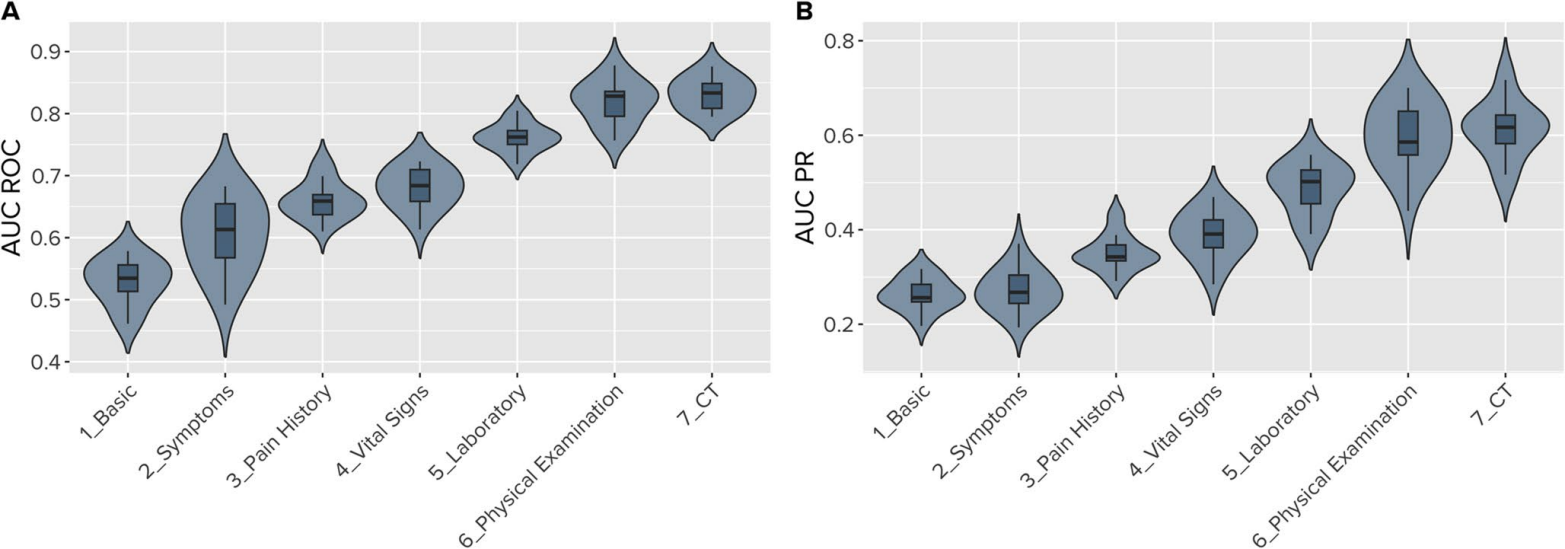
Violin-/Boxplots showing the performance at each clinical timepoint (aka feature sets). **A** Area under curve (AUC) for the receiver operating (ROC) curve and **B** AUC for the precision recall (PR) curve

Across all iterations, the final model incorporating all feature sets achieved a median AUC ROC of 0.83 (IQR 0.81–0.85) and a median AUC PR of 0.62 (IQR 0.58–0.64) (Fig. 1). Performance increased stepwise with the addition of feature sets (Fig. 2; Table 3). Models based solely on demographics performed poorly (AUC ROC 0.53, AUC PR 0.26). The addition of symptoms improved discrimination modestly (AUC ROC 0.61). Predictive accuracy increased with pain history (AUC ROC: 0.66, AUC PR: 0.34) and vital signs (AUC ROC: 0.68, AUC PR: 0.39). Substantial gains were achieved after laboratory values (AUC ROC 0.76, AUC PR 0.50). Physical examination yielded near-final performance (AUC ROC 0.83, AUC PR 0.59), which only marginally improved by adding CT findings (AUC ROC: 0.83, AUC PR: 0.62). Given the class imbalance in our cohort, AUC PR values were lower than AUC ROC and should be interpreted relative to the positive class prevalence.

**Table 3:** Median (IQR) performance at each clinical timepoint (aka feature sets). Area under curve (AUC) for the receiver operating (ROC) curve and AUC for the precision recall (PR) curve

	AUC ROC	AUC PR
1. Basic	0.53 (0.51–0.56)	0.26 (0.25–0.28)
2. Symptoms	0.61 (0.57–0.65)	0.27 (0.24–0.30)
3. Pain History	0.66 (0.64–0.67)	0.34 (0.33–0.37)
4. Vital Signs	0.68 (0.66–0.71)	0.39 (0.36–0.42)
5. Laboratory	0.76 (0.75–0.77)	0.50 (0.46–0.53)
6. Physical examination	0.83 (0.80–0.84)	0.59 (0.56–0.65)
7. CT	0.83 (0.81–0.85)	0.62 (0.58–0.64)

### Feature importance

**Fig. 3:**
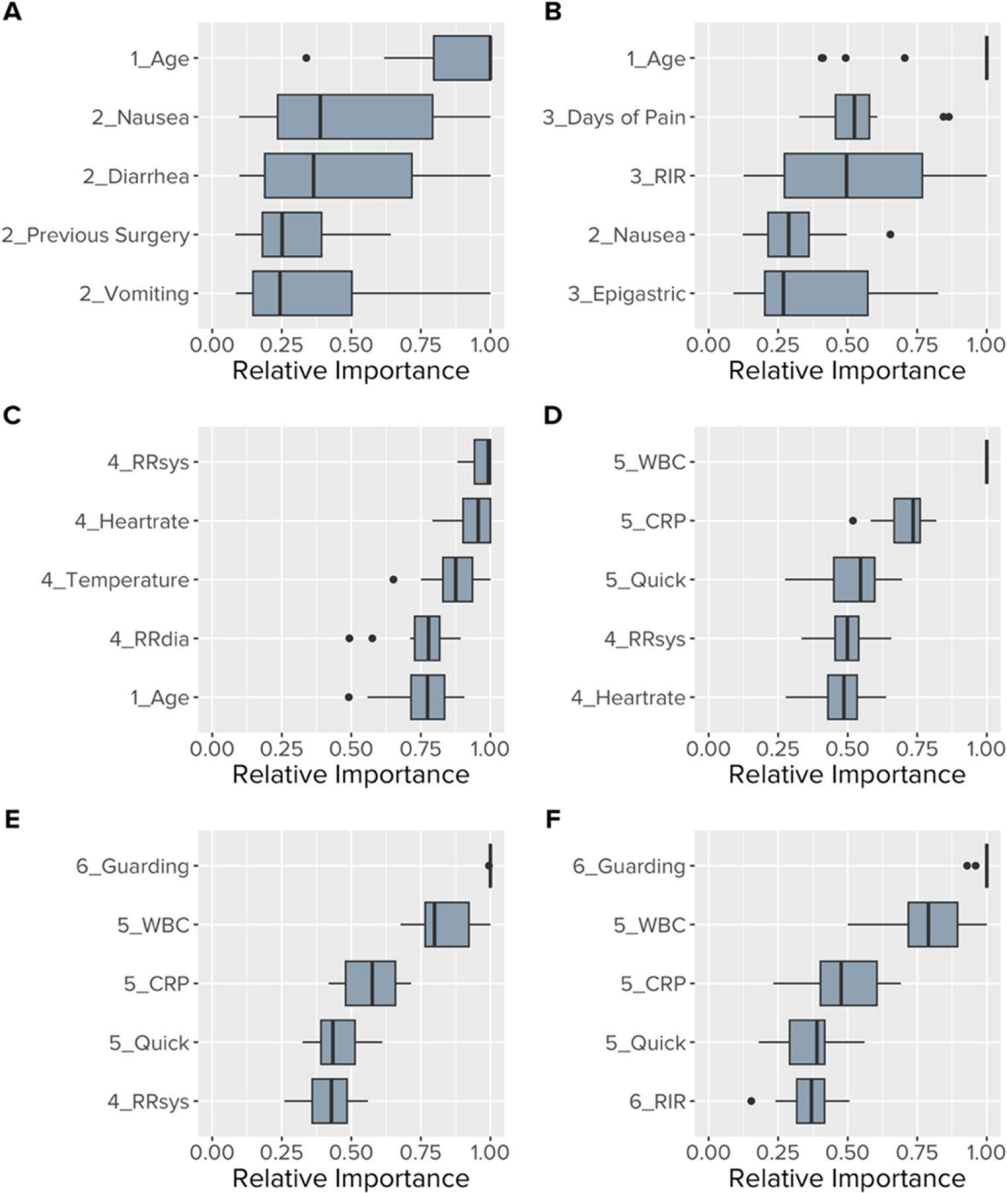
Boxplots for feature importance (top5) for different feature sets. **A** 2_Symptoms **B** 3_Pain History **C** 4_Vital Signs **D** 5_Laboratory **E** 6_Physical Examination **F** 7_CT

Permutation-based feature importance analyses demonstrated a shift in the relative contribution of predictors across feature sets (Fig. 3). In the early phase (symptom set, Fig. 3A), the most influential features were age, nausea, diarrhea, previous surgery, and vomiting. With the addition of pain history (Fig. 3B), age and nausea remained relevant, while pain duration and pain location emerged as leading predictors. When vital signs were incorporated (Fig. 3C), they became dominant, with age as the only early feature still ranking among the top five. After laboratory values were included (Fig. 3D), markers of inflammation and organ function, particularly C-reactive protein (CRP), white blood cell count (WBC), and prothrombin time (Quick), contributed most strongly to model predictions. These variables maintained their importance in larger models that included physical examination and CT (Fig. 3E and F). At that point, only guarding and tenderness in the right iliac region supplemented the predictive value of the laboratory markers.

### Global interpretability

**Fig. 4:**
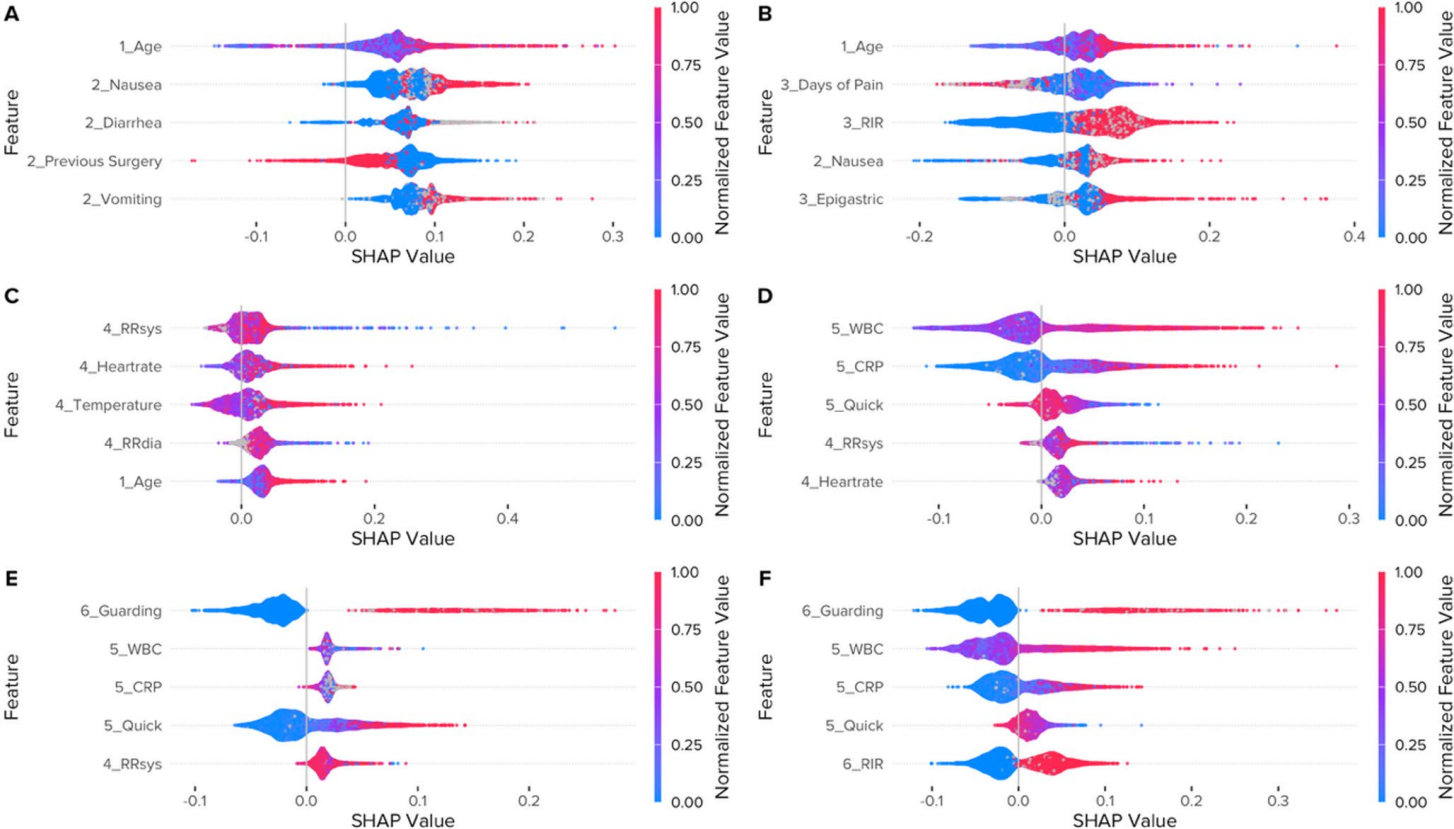
SHAP summary plots displaying the impact of individual features (top5) on the model’s output for different feature sets. **A** 2_Symptoms **B** 3_Pain History **C** 4_Vital Signs **D** 5_Laboratory **E** 6_Physical Examination **F** 7_CT

SHAP analyses provided complementary insights into feature contributions across the diagnostic path (Fig. 4). In the symptom set (Fig. 4A), nausea and vomiting were strongest contributors alongside age. After addition of pain history (Fig. 4B), pain duration and location emerged as important features. With vital signs (Fig. 4C), systolic blood pressure and heart rate dominated the model. In the laboratory set (Fig. 4D), WBC, CRP, and Quick were the leading contributors. After physical examination was added (Fig. 4E), guarding became influential alongside laboratory markers. With CT included (Fig. 4F), laboratory variables remained dominant, and CT findings did not appear among the top five contributors. Across all stages, SHAP plots consistently showed that abnormal values of laboratory and clinical variables had been associated with higher predicted risk, while normal ranges or absent findings had been linked to non-operative outcomes.

### Local interpretability

**Fig. 5:**
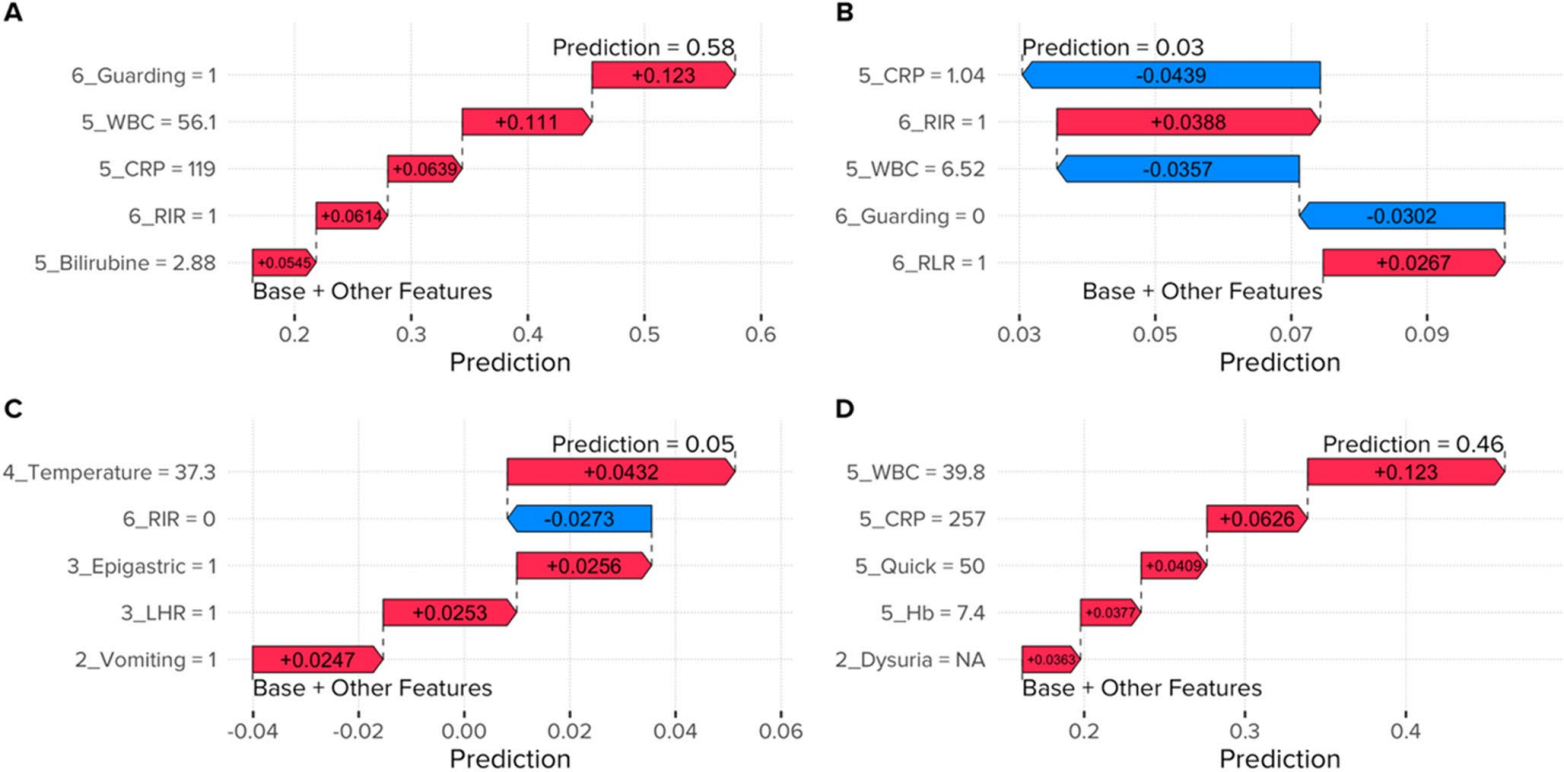
SHAP Waterfall Plots Illustrating Feature Contributions to Predictions for Four Individual Cases. Each panel (**A** acute appendicitis – outcome: urgent surgery, **B** unspecific abdominal pain – outcome: no surgery, **C** unspecific abdominal pain – outcome: no surgery, **D** liver abscess with sepsis – outcome: no surgery) represents a different patient case, showing how specific clinical features (e.g., lab values, physical exam findings) contribute to the model’s final prediction. Red bars indicate features increasing the predicted probability, while blue bars indicate features decreasing it. The numerical value on each bar quantifies the magnitude of that feature’s impact

SHAP waterfall plots illustrated how individual predictions were composed (Fig. 5). In one representative patient with acute appendicitis, high CRP, elevated bilirubin, and the presence of guarding were the strongest contributors to a high predicted probability of urgent surgery (Fig. 5A). Another patient with a liver abscess and sepsis also received a high predicted probability, driven by abnormal laboratory markers and systemic signs of infection (Fig. 5D). In contrast, patients with non-specific abdominal pain were assigned low probabilities of surgery, explained by normal laboratory values and absence of clinical signs (Fig. 5B and C).

## Discussion

Our study demonstrates that ML prediction could support surgical CDM in patients presenting with AAP, achieving clinically relevant discrimination already at an early stage of the diagnostic pathway. Although early recognition of urgency remains difficult, it is associated with lower morbidity and shorter hospitalization [14, 15]. This phase typically occurs within the first 60 min after presentation, when clinical assessment and laboratory results become available. At this point, clinicians are required to make time-critical decisions under diagnostic uncertainty [15]. In this context, our model’s performance increased stepwise, with near-final discrimination reached after laboratory values and physical examination. Therefore, our findings suggest that ML-based prediction could be deployed at this critical stage to support early triage, reducing diagnostic uncertainty and enabling timelier and more targeted diagnostic and therapeutic CDM. Importantly, our model is designed as an explainable decision-support tool. Beyond performance metrics, interpretability analyses showed that predictions had been consistently driven by clinically plausible features, both at the population and patient level. Using SHAP-based methods, predictions can be interpreted at the patient level by identifying the most influential contributing features. This enables clinicians to understand and critically appraise the outputs and thereby facilitates reliance and integration into clinical routine. This is particularly relevant as recent reviews of AI-enabled decision support in surgery emphasize explainability as a key requirement for clinical adoption, which remains insufficiently addressed in many existing models [9, 16].

### Role of CT

A central decision point in the diagnostic pathway is the transition from clinical assessment to radiological work-up, where the key question is whether urgent treatment within 24 h is required [5, 11]. In our stepwise analysis, clinically relevant discrimination was achieved with near-final performance after laboratory testing and physical examination and before the inclusion of CT features. Importantly, our CT feature set was deliberately restricted to four binary features representing typical surgical pathologies. In clinical practice, however, CT provides substantially more detailed information regarding differential diagnosis, disease characterization, and treatment planning. Our simplified representation may therefore underestimate the full clinical contribution of CT beyond the binary decision for or against urgent surgery. Previous studies provide complementary perspectives on this decision point. Schulwolf et al. studied more than 4,000 patients with small bowel obstruction and found no additional discriminative benefit from CT when predicting the need for surgery within 24 and 48 h [17]. Conversely, a multicenter diagnostic-accuracy study demonstrated that clinical assessment without imaging support is associated with higher rates of missed urgent conditions and false positives [11]. CT therefore remains an important diagnostic tool, as the absence of imaging may lead to overtreatment of non-urgent conditions (e.g., diagnostic laparoscopy), and surgeons rank CT among the most relevant modalities for assessing surgical urgency [9]. Moreover, a retrospective study demonstrated that shorter CT turnaround times are associated with reduced emergency department and hospital length of stay as well as lower healthcare costs [18]. From a resource perspective, universal CT is neither feasible nor desirable due to radiation exposure and costs. In this context, our findings extend the evidence by showing that ML-based prediction could guide early triage, ensuring that limited scanner capacity is prioritized for patients at highest risk and enabling more targeted therapy for critically ill patients [19]. In addition to CT, ultrasound is frequently used as a first line imaging modality in the work-up of AAP, but its hypothesis-driven and operator-dependent nature limits its standardization and comparability across patients [5, 15, 20]. Future work may explore the integration of standardized ultrasound features to further improve model performance while maintaining robustness and generalizability.

### Role of laboratory testing

Our findings also highlight the importance of laboratory testing in early triage. At the pre-imaging stage, timely and comprehensive laboratory diagnostics were essential for model performance, with CRP and WBC consistently among the most influential predictors across multiple timepoints. This contrasts with the perception of German surgeons, who ranked laboratory values only fifth among seven diagnostic modalities, below vital signs and pain history [9], and with guideline statements that CRP and WBC alone are insufficient to distinguish urgent from non-urgent conditions [5]. Notably, existing guidelines for AAP were developed without consideration of AI or ML and therefore do not reflect the potential of these methods to leverage laboratory and clinical features in a structured, reproducible way. Such discrepancies may partly reflect differences in study endpoints: while prior studies evaluated urgent treatment, we focused on urgent surgery, which represents a narrower but clinically decisive outcome. Importantly, biomarkers not included in our study may further improve predictive accuracy. For example, interleukin-6 has been shown to outperform CRP, procalcitonin, and WBC in differentiating urgent from non-urgent abdominal conditions [21]. Taken together, laboratory markers contribute meaningfully to the prediction of urgent surgery but cannot replace radiological imaging or surgical examination in CDM.

### Role of the physician/surgeon

We deliberately placed the physical examination at the end of the diagnostic pathway, although this diverges from the bedside workflow. Up to feature set 6, our model relied entirely on structured anamnesis, vital signs, and laboratory testing, indicating that predictive performance can be achieved before a surgical specialist becomes involved. This is particularly relevant given the shortage of surgical workforce in emergency departments and highlights the potential of decision support in resource-constrained settings [22]. This raises the question whether software can support a structured work-up and provide consistent triage criteria. Such support may be particularly valuable during emergency-department overcrowding, which is known to worsen patient outcomes [23]. In this context, ML-assisted triage could help identify patients who require urgent escalation, while allowing others to be safely deferred. These considerations are particularly relevant in the light of different international care models. In Anglo-American systems, emergency physicians often act as the primary decision-makers, while in Germany surgical consultation is typically obtained earlier. Adequate predictive performance before surgical contact suggests that ML could act as a bridge: supporting non-surgical physicians in gatekeeping roles, while also providing surgeons with transparent reasoning once they are involved. Nevertheless, the surgeon’s role remains central. German surgeons ranked physical examination as the single most important modality for deciding whether urgent surgery is required [9], and in our data its inclusion improved AUC PR more than AUC ROC, consistent with a potential reduction in false positives. Clinically, this is plausible: guarding was consistently among the top predictors overall and represents a cardinal sign of peritonitis. However, recognition and interpretation of guarding are inherently clinical acts that require surgical expertise, standardized definitions, and experience. Beyond diagnostic accuracy, surgeons carry responsibility for explaining diagnostic reasoning and treatment strategies to patients, families, and colleagues. This underscores an ethical distinction: while transparent, validated AI tools can serve as functional aids, they cannot be trusted in the moral sense because they lack accountability and responsibility [24, 25]. Trust remains reserved for the physician, whose judgment and accountability must not be replaced.

### Technical considerations

Our dataset contained missing values that were not at random. For example, CT variables are absent when no scan was performed, which could implicitly suggest the absence of findings such as free air, though the true rate remains unknown. Similarly, there may be a bias toward documenting only pathological findings, such that missing values in binary features effectively function as “no.” While median or mode imputation is intuitive for clinicians, missing data imputation remains a matter of debate in ML studies. We deliberately avoided imputation to reduce the risk of data leakage and because the H2O random-forest framework can internally accommodate missingness [12, 13]. Robustness was enhanced by repeating the training and evaluation procedure in multiple iterations. These methodological choices are consistent with recent state-of-the-art recommendations for AI-enabled decision support in surgery, which emphasize transparency, avoidance of leakage, and robust validation [16].

### Limitations

Nonetheless, important limitations must be acknowledged. Its retrospective, single-center design limits generalizability. Preselection of surgically managed patients introduces a selection bias, excluding internal medicine, urological, and gynecological causes of AAP. Conducted in a tertiary referral center, the cohort may overrepresent severe cases. At the same time, the tertiary setting provides a large, heterogeneous population of surgical emergencies and input from multiple treating physicians. The chosen endpoint of urgent surgery within 24 h is clinically robust, yet narrower than the “urgent treatment” definition used in some prior studies, which complicates direct comparison with guidelines. An alternative endpoint such as the true need for urgent surgery would be of high clinical and scientific relevance but is difficult to define reliably in retrospective emergency surgical cohorts and prone to interpretation and hindsight bias. Methodologically, we evaluated only random forests, which provide robustness and interpretability, but other algorithms may yield different performance. Laboratory features were restricted to routine markers; advanced biomarkers such as interleukin-6 or structured radiological data beyond standard reports were not included. Interpretability analyses using permutation importance and SHAP provided valuable insights, but they remain associative and should not be mistaken for causal inference. Finally, the model was evaluated retrospectively and outside the clinical workflow. Prospective validation, integration into real-time CDM, and assessment of acceptance among clinicians and patients remain essential steps before clinical application.

## Conclusion

This study demonstrated that interpretable ML could provide valuable insights into the diagnostic process of AAP. The dynamic shifts in feature relevance across clinical timepoints highlight that prediction is not static but evolves alongside the diagnostic trajectory. Importantly, the consistent presence of both subjective symptoms and objective biomarkers among the most influential predictors underscores the multidimensional nature of CDM in emergency surgery. Rather than replacing physicians, these findings emphasize the irreplaceable role of physical examination and clinical judgment, while ML can serve as a transparent and supportive tool. Prospective evaluation in real-world clinical workflows is warranted to validate these results and to ensure that algorithmic support enhances, rather than supplants, established diagnostic practice.

## Data Availability

The data that support the findings of this study are not openly available due to reasons of sensitivity and are available from the corresponding author upon reasonable request.
